# The Kinematics of Cytotoxic Lymphocytes Influence Their Ability to Kill Target Cells

**DOI:** 10.1371/journal.pone.0095248

**Published:** 2014-05-06

**Authors:** Purnima Bhat, Graham Leggatt, Klaus I. Matthaei, Ian H. Frazer

**Affiliations:** 1 The University of Queensland Diamantina Institute, Translational Research Institute, Brisbane, Queensland, Australia; 2 Medical School, The Australian National University, Canberra, Australian Capital Territory, Australia; 3 The John Curtin School of Medical Research, The Australian National University, Canberra, Australian Capital Territory, Australia; Leiden University Medical Center, Netherlands

## Abstract

Cytotoxic lymphocytes (CTL) have been reported to show a range of motility patterns from rapid long-range tracking to complete arrest, but how and whether these kinematics affect their ability to kill target cells is not known. Many *in vitro* killing assays utilize cell lines and tumour-derived cells as targets, which may be of limited relevance to the kinetics of CTL-mediated killing of somatic cells. Here, live-cell microscopy is used to examine the interactions of CTL and primary murine skin cells presenting antigens. We developed a qualitative and quantitative killing assay using extended-duration fluorescence time-lapse microscopy coupled with large-volume objective software-based data analysis to obtain population data of cell-to-cell interactions, motility and apoptosis. *In vivo* and *ex vivo* activated antigen-specific cytotoxic lymphocytes were added to primary keratinocyte targets in culture with fluorometric detection of caspase-3 activation in targets as an objective determinant of apoptosis. We found that activated CTL achieved contact-dependent apoptosis of non-tumour targets after a period of prolonged attachment – on average 21 hours – which was determined by target cell type, amount of antigen, and activation status of CTL. Activation of CTL even without engagement of the T cell receptor was sufficient to mobilise cells significantly above baseline, while the addition of cognate antigen further enhanced their motility. Highly activated CTL showed markedly increased vector displacement, and velocity, and lead to increased antigen-specific target cell death. These data show that the inherent kinematics of CTL correlate directly with their ability to kill non-tumour cells presenting cognate antigen.

## Introduction

The skin is a very tolerant organ. It forms a primary barrier against environmental insults and is colonized by a large array of microorganisms against which it does not mount an immune response. KC have been shown to be key players in mediating the tolerant state of skin, strongly suggesting that the relationship between cytotoxic CD8^+^ T cells and KC targets may be unique and complex.

Cytotoxic CD8^+^ T cells are highly dynamic once activated. They have been shown to rapidly traffic in tissue in response to cytokine with a primary function to find and kill target cells expressing cognate antigen on their surface. These cells are certainly capable of killing KC targets *in vitro* [1], [2] and have been shown to effect skin graft rejection *in vivo* [3]. Activated T cells were shown *in vivo* to traffic to inflamed skin, even in the absence of cognate antigen [4]. The ability of a CD8^+^ T cell to move and seek out antigen presenting cells and target cells is clearly important to their cytotoxic function, however there are few studies examining the relationship between the kinematics of CD8^+^ T cells and their ability to kill non-tumour targets. We use the term kinematics to specifically to refer to the local motility and dynamic behavior of CD8 T cells as distinct from T cell traffic into and out of tissues.

CD8^+^ T cells kill via a variety of well-described mechanisms including through perforin and granzymes via direct cell-to-cell contact with targets at the immunological synapse, interaction of Fas-ligand to bind with Fas that may or may not require direct contact or proximity to target cells, and via release of cytokines such as IFN-gamma or TNF-alpha that may induce apoptosis in target and bystander cells. Which mechanisms prevail in a particular circumstance are unclear, but tissue-dependent and possibly signal dependent factors likely play an important role and account for some of the variability in T cell effector responses.

Despite their significance in skin disease, it is not known how CD8^+^ T cells kill KC. Traditional killing assays, although providing valuable quantitative killing data *in vitro* using ^51^chromium labeling of target cells, or *in vivo* using adoptive transfer of target/effector cells, fail to inform about the motility and cell-to cell interactions during the killing process. Additionally, these assays often use as targets tumour cell lines that express antigen in vast excess and fail to differentiate, compromising their physiologic relevance.

The ability to visualize T cell behavior in vivo in mouse models by microscopy has revealed how remarkably dynamic these cells are in their physiologic environment [5]. It has been remarkably difficult, however to visualize the process of *in vivo* cell death directly, possibly because the process is very slow, and most *in vivo* imaging platforms can only visualize one static region for short periods of time. Using multiple 35 min imaging periods, CTL killing of highly antigenic induced tumours *in vivo* was estimated to be over 6 hours [6]. In non-tumour models where antigen expression is more variable, in vivo imaging has suggested that the process is likely to be even slower [7].

Time-lapse microscopy has been used to examine CTL behavior in a variety of target killing settings, revealing important fundamental mechanisms of immune mediated killing [8] [9]. Most of these microscopy studies examined only few cells per well and were dependent on subjective examination, thus they do not provide cell population data. Recently, low-magnification imaging used to examine larger numbers of NK cell killing showed that non-primary effectors killed non-primary target cells almost exclusively by sequential interactions [10]. Similar imaging using primary NK cells showed that the serial killing – also of non-primary targets – was performed by only a specific subgroup of the all of the NK [11]. While the use of live microscopy has advanced our understanding of target-effector interactions, these studies also highlight the differences between the behavior of primary and non-primary cells in this system.

We developed a quantitative and qualitative killing assay using sustained-duration live-cell time-lapse microscopy and used this to answer fundamental questions about the motility behavior of primary cytotoxic CD8^+^ T cells in relation to their ability to kill primary keratinocytes presenting low levels of cognate antigen.

## Materials and Methods

### Mice

B6.K5mOva (K5mOva) mice were a kind gift from W.R. Heath, WEHI, Australia. OT-1 mice were purchased from the Animal Resource Centre, Perth, Australia. B6.Nzeg mice, express enhanced green fluorescent protein (EGFP) from the ubiquitously expressed CMV early enhancer/chicken beta actin promoter driving a nuclear localization signal containing EGFP on a C57BL/6 genetic background. These were bred with OT-1 mice to create OT-1.Nzeg mice. All CD8^+^ T cells were derived from these EGFP strains and will be referred to as “OT-1” or “B6” mice.

### Ethics statement

Experiments were formally approved in writing by the University of Queensland animal ethics committee (UQDI/290/11/NHMRC/NIH (NF)). All mouse sacrifice was performed as recommended by CO_2_ inhalation or decapitation, and all efforts were undertaken to minimize suffering to the animals.

### Reagents

The following anti-mouse antibodies were purchased from BD Pharmingen: anti-CD44, anti-IFN-gamma, anti-CD8, relevant isotype antibodies. Anti-CD62L was from eBioscience. Red SR-FLIVO was courtesy of Immunochemistry laboratories. Z-DVED-FMK was purchased from Sigma. Recombinant IFN-gamma was purchased from R&D Systems. Ovalbumin (OVA) was purchased from Sigma. All media for cell culture and IL-2 was purchased from Invitrogen.

### Keratinocyte harvest and culture

Primary KC were harvested from 3–4 day old mice as described previously [1]. Briefly, skins were removed from sacrificed mice, and digested overnight at 4°C in 10% dispase solution before brief trypsinization. Resuspended KC were seeded in DMEM with 5% foetal calf serum, onto 24-well tissue culture plates. Media was changed to Serum-Free Medium with supplements 24 h later. We imaged cells at 60–70% confluence, usually 48 hours after plating, when KC motility was limited by contact with neighbouring cells.

### Activation and harvest of CD8^+^ T cells

Mice were immunised by subcutaneous injection with OVA (50 µg) and Quil A (20 µg). Seven to ten days later, splenocytes were harvested as previously described [3]. For T cell selection, cells were stained with anti-CD8^+^ antibody, and 1 µg propidium iodide (Invitrogen) before cell sorting to obtain >98% pure population of cells (Moflo).

### Co-culture

**Figure 1:**
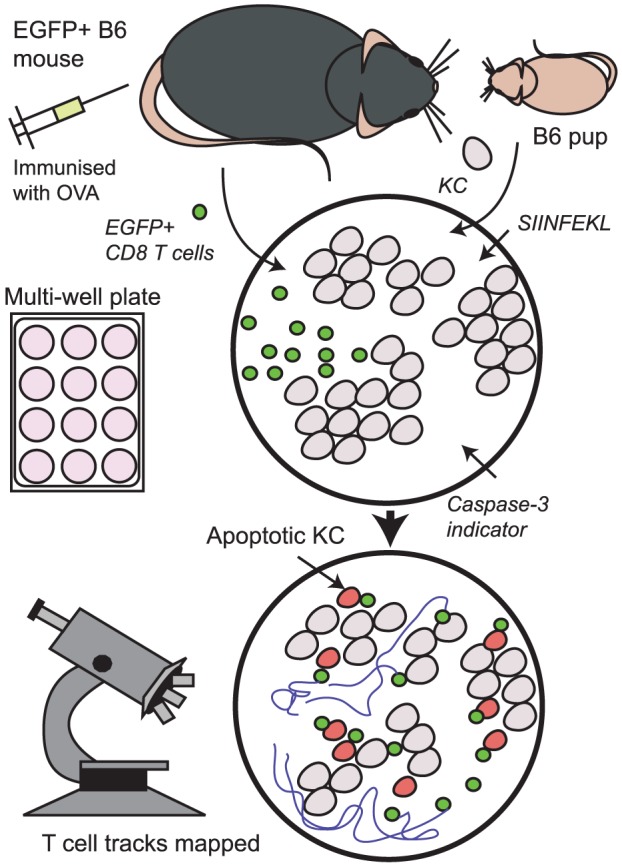
Killing assay. Primary KC from B6 mouse pups were cultured in multi-well plates until 70% confluent, and then loaded with SIINFEKL peptide. Antigen-specific CD8^+^ T cells from EGFP^+^ B6 mice were isolated and co-cultured with target cells in the presence of cell-permeable caspase-3 indicator dye and imaged by time-lapse fluorescence microscopy or by confocal microscopy every 12 minutes for 30 h. Cell death was noted by colour change, morphology, and behaviour.

(Fig. 1) We added 1 µg.ml^−1^ SIINFEKL peptide (AusPep, Australia), comprising amino acids 257–264 of OVA, to KC for 1 h at 37°C, and washed 3 times in PBS to remove non-incorporated peptide. We added 5×10^3^ CD8^+^ T cells per well into duplicate wells, aiming for a target:effector ratio of approximately 1∶1. Cells were imaged at 37°C with 5% CO_2_ for 30 h in media comprising 45% RPMI, 5% foetal calf serum, 50% serum-free medium, 5 ng.ml^−1^ IL-2 and 2% SR-FLIVO solution. Each plate included negative control wells: co-cultures without peptide, and KC monocultures.

### Cell killing assay

Red SR-FLIVO, a cell-permeable indicator dye that fluoresces red upon activation of caspase-3, was added to the media of target-effector co-cultures, and colour change was denoted the point of apoptotic death. As cell division was a rare event, death rate was calculated as number of red cells >12 µm diameter (to exclude T cells) as a percentage of total number of KC in the field at time 0. Averages were from 3 fields per well of duplicate wells from at least 3 replicate experiments.

### Microscopy

Confocal microscopy was performed on a Zeiss Meta-250 confocal microscope using Zen software, with ×25 objective lens. Cells were grown in a coverslip-based chamber and five 2 µm Z-sections from ten fields of view were imaged for 30 hours. Fluorescence microscopy is susceptible to significant phototoxicity, amplified by the addition of fluorophores and visible in our system as keratinocyte cell membrane damage and swelling. We acquired serial images at different rates from 2 min to 15 minutes apart and compared the average track velocity, and displacement, calculated over the total duration of imaging. Note that as these are *vectorial* calculations, they are determined by position of a particle at time when movement stops in relation to the position at time 0 (Displacement). Velocity is the displacement divided by time taken for the cell to reach its final position. The average displacement and velocity of the T cells tracks was not affected by more frequent imaging, however there was markedly more phototoxicity with frequent exposures. As it appeared that target killing was a slow process *in vitro* requiring prolonged co-culture of over 30 hours, an optimal imaging frequency of 12–15 min was determined for this level of magnification without loss of dynamic detail, but with no detectable phototoxicity. This method of calculation will therefore not account for changes in speed of the cell during imaging, and will not account for a cell moving very fast but in a convoluted track. Interestingly, displacement and actual travel distances were very closely correlated in these experiments, but as speed calculations in the latter were dependent on frequency of imaging, the vector calculation was utilized instead.

To investigate large numbers of interactions, low-power magnification with time-lapse microscopy was performed on a Zeiss Axiovert 200 M microscope. Images were acquired from five fields of view per well from duplicate wells using a ×10 objective lens for 30 hours, providing quantification of around 200 target cells per field of view. Cells were maintained with humidification, in 5% CO_2_, at 37°C during imaging.

### Image analysis

**Figure 2:**
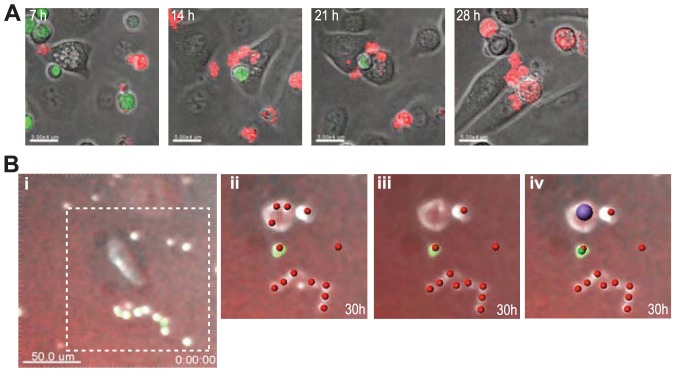
Imaging of CTL and KC targets. **A**. Series of still confocal microscopic images from Movie S1 of a single field over 30 hours. CD8^+^ T cells (green) attach to KC after 7 h of co-culture and cause apoptosis (red) at 21 h. Overlay images were from brightfield and fluorescence channels, acquired using a ×25 objective lens, mean intensity projection of a Z-stack of 5 confocal images of 2 µm each. (Bar, 50 µm) **B**. Analysis of epifluorescent imaging of CTL and KC co-cultures by software spot detection. Merged views of brightfield, rhodamine filter and GFP filter are shown in each frame at time 0 (i) and at 18 h (ii–iv). i. At t = 0, a KC and several smaller green T cells can be seen. ii. Spot detection without correction, using parameters: Red^+^, size 7 µm. iii. Spot detection after manual correction for adjacent spots. iv. Spot detection using parameters: Green^+^, size 7 µm (green); red channel+, size 7 µm (red); and including Red^+^, size 15 µm (purple).

We used Imaris 7.6.3 software, with MATLAB extension, to de-convolute and analyse images, and generate movies. Analysis was performed on raw images. Parameters were set using control images and saved settings applied to all data within an experiment for consistency. T cells were identified using the software wizard by size (7 µm) and EGFP expression, manual adjustment of quality, filtering out spots within 10 µm of the edge, and tracked through each frame with maximum gap of 1, distance of 25 µm using the built-in algorithm. Spot selection was examined manually and corrections applied as required on each final frame per section. T cell death was calculated by spot selection in the red channel based on size (7 µm) and manually corrected particularly adjacent spots if they were touching, and also where multiple small spots had been assigned to a single large KC spot (Figure 2B, Movies S8–S10).

#### T cell tracking

T cells were identified using the software by size and EGFP expression, and tracked through each frame using the built-in algorithm according to the parameters above. Tracks were manually examined and corrected as required. Typically five to seven tracks per movie required changes, comprising around 2% of the tracks. Imaris ended tracks when the particle could no longer be found in the green channel, indicating T cell death or movement out of the field of view.

Cells moving out of the field of view were excluded manually. It is possible that this excluded cells with very long travel displacements, affecting both net displacement and velocity results. We note that this happened rarely where travel distances were very small as with Naïve T cells, or in the absence of cognate antigen loading, but around 2–5% of cells were discarded in other wells.

#### Cell death

While the EGFP was very bright and easily distinguishable from background, the signal:noise ratio in the red channel indicating apoptosis was lower, and varied between experiments. For each experiment, control wells were carefully examined and a minimal threshold set for determination of apoptosis. If the cell became red during the movie, it was determined that it was apoptotic at that point even if the red signal became dimmer during the rest of filming as apoptosis is considered to be an irreversible event. For example, in Movie S1, apoptosis was considered to have occurred at around 14 hours.

Within the red channel, keratinocyte death was determined by spot selection based on size (approximately 17 µm). Movies were divided into 5-hour intervals and the number of new spots in the final frame of each time interval was determined by Imaris. It was not possible looking at a KC-sized red spot to accurately determine whether this cell had been a KC or a fibroblast. Fibroblasts contaminating each field of view were identified at time 0, and de-selected from the final analysis. As heavily contaminated cultures were not used in the experiments, in any one field, there were less than 5 fibroblasts.

Data were exported into Excel and into Graphpad Prism for statistical analysis. Typically, over 150–250 CTL and 80–100 primary KC per experiment were analysed in this way from duplicate wells. A 3×3 median filter and minor contrast adjustments, and cropping were performed using Imaris on example still images and movies for publication.

### Statistics

Cell culture data were collected from duplicate wells. All experiments were repeated a minimum of three times, and technically successful experiments were all included in the statistical analysis. N numbers given in each figure represent the number of independent experiments performed to obtain the data shown. Within each experiment, replicate wells were used and the data averaged to give one value per experiment. Each dot in a scatter plot refers to one experiment, unless specifically stated in the legend. We used the one-tailed Mann-Whitney test, or one-way ANOVA to compare non-parametric data. Data sets were considered significantly different if p<0.05.

## Results

### CD8^+^ T cells require direct prolonged contact with target cells to kill KC *in vitro*

We developed a live-cell assay to study the kinetics of effector cell travel, attachment, and killing.

Cultured primary KC from B6 pups were loaded with SIINFEKL, the T cell epitope of OVA, before adding CD8^+^ T cells isolated from EGFP^+^OT-1 mice, a B6-derived transgenic mouse in which T cells express the TCR specific for SIINFEKL peptide (Fig. 1). Each experiment included the following controls imaged concurrently – KC monoculture, co-culture without peptide. Thus we could collect data from a large numbers of cells (over 100 KC and CTL per well per experiment) and compare between experiments. In all experiments that were technically successful, KC death without T cells was <7% and KC death in the absence of cognate peptide was <10% (Movies S2–S4).

**Figure 3:**
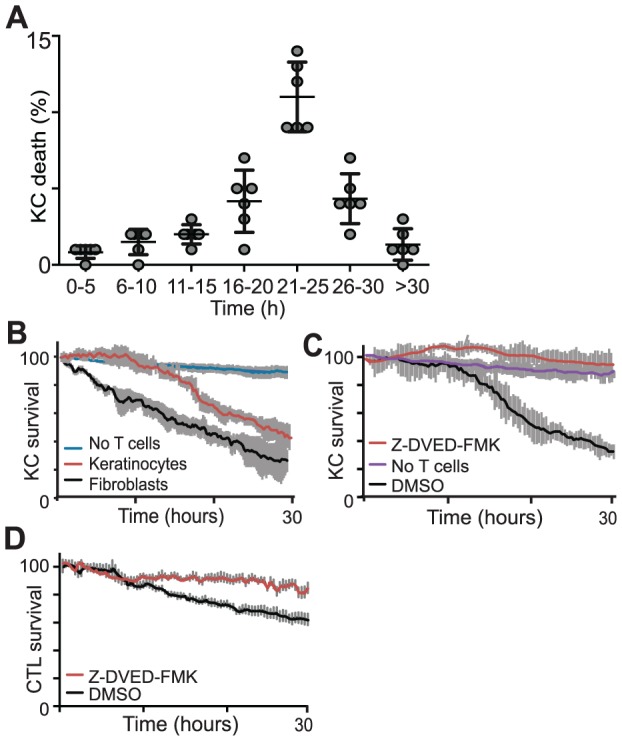
CD8^+^ T cells require direct prolonged contact with target cells to kill KC. Primary KC loaded with ovalbumin peptide were co-cultured with CD8**^+^** T cells from EGFP^+^OT-1 mice and imaged over a 30-hour period in the presence of a cell-permeable indicator dye that fluoresces red upon activation of caspase-3. **A**. The number of caspase-3 positive keratinocytes in each 5 hour time period per experiment was determined by standardized objective analysis of movies by specialized imaging software. N = 6, error bars are SEM. **B**. In primary KC culture, contaminating fibroblasts are readily distinguished by their morphology. These also uptake peptide and present as targets for CTL. KC and fibroblast survival in co-culture with OT-1 cells; (Pooled results N = 5, error bars are SD). **C**. Addition of caspase-3 inhibitor, Z-DVED-FMK, an inhibitor of caspase-3, resulted in abrogation of CTL-mediated apoptosis. (Pooled results N = 5, error bars are SD). **D**. T cell death, following a similar pattern to KC death, is abrogated by the addition of Z-DVED-FMK (Pooled results N = 3, error bars are SD).

CD8^+^ T cells attached to target KC presenting SIINFEKL within 5–7 hours, however it was more than 15 h later (average 21 h) that target cell death ensued (Fig. 2A & B, Fig. 3A). These attachments were mostly single target-effector interactions leading to both KC and T cell death (Movies S1, S5, S7). Direct and usually prolonged effector-KC target contact was required universally to achieve death by apoptosis. We note there was a 5–7% baseline KC death rate during culture, determined by KC mono-culture (Fig. 3C, Movie S4). Primary skin cultures usually contain a small number of fibroblasts – readily distinguishable from KC by their morphology – which show minimal death at passage 0 even though the culture conditions were not designed for their growth. CD8^+^ T cell killing of these fibroblasts in the primary skin cell culture was a convenient cell-type control. Fibroblast target deaths displayed linear kinetics, with no lag period (Fig. 3B), indicating that the prolonged attachment prior to death was a characteristic specific to target cell type: keratinocytes.

Activation of FLIVO-red strongly indicated that CD8^+^ T cell mediated KC apoptosis was caspase-3 dependent. To investigate this further, we incubated the effector-target co-cultures with a specific caspase-3 inhibitor, Z-DEVD-FMK, or dimethyl sulphoxide (DMSO) as control. Z-DEVD-FMK abrogated KC death, death of sparsely found fibroblasts, and also T cell death in co-culture as evidenced by cell morphology, behaviour (division, locomotion) and lack of colour change (caspase-3 activation) (Fig. 3C,D). We surmise that CD8^+^ T cells kill KC solely by caspase-dependent mechanisms.

### Activation of CD8^+^ T cells is sufficient to enhance movement kinematics

**Figure 4:**
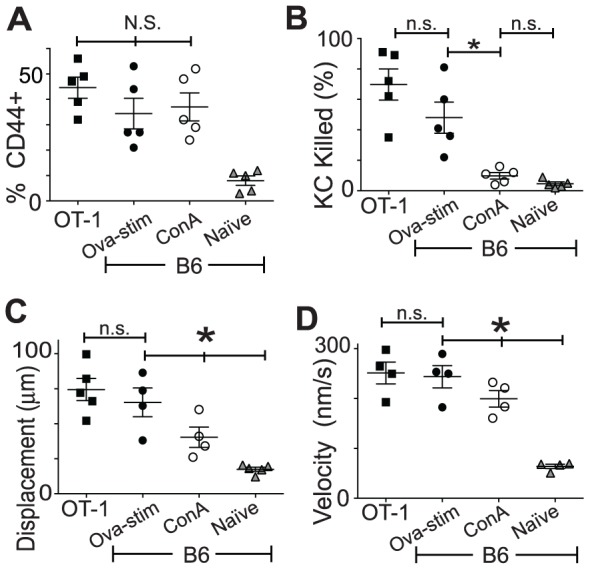
CD8^+^ T cell activation is sufficient to promote enhanced kinematics. CTL from OT-1 mice (OT-1), or from B6 mice either immunized with OVA and stimulated with SIINFEKL (Ova-stim), or stimulated with concavalin A (ConA), or not stimulated (Naïve), were added to KC cultures loaded with SIINFEKL and imaged for 30 h. **A**. CTL were isolated by FACS based on size and granularity and by EGFP and CD8 expression, and stained for CD44. Percentage of isolated cells that are CD44^+^ are shown. **B**. Percentage of total KC that were killed after 30 hours of co-culture with different groups of CD8+ T cells. KC killing was determined by red fluorescence change indicating activation of caspase-3. **C**. Vector displacement and velocity (**D**) of the effector cells added to antigen-presenting KC over 30 hours of imaging. (A–D N = 5, error bars SEM) (n.s. not significant, *p<0.05; N.S. no significant difference between indicated groups, *p<0.05 between indicated groups ANOVA.).

We investigated the movement kinematics of CD8^+^ T cells in the presence of antigen-presenting target cells. CD8^+^ T cells were isolated from EGFP^+^B6 mice 10 days after immunization with OVA peptide and cultured for 3 days with SIINFEKL and IL-2. Naïve splenocytes from EGFP^+^B6 mice were cultured with IL-2, with some receiving stimulation with concavalin A overnight. CD8^+^ T cells were also isolated from EGFP^+^OT-1 mice. T cell populations showed similar percentages of CD8^+^CD44^+^ T cells (Fig. 4A) indicating similar levels of activation. However, only OVA-stimulated T cells were able to effect target cell killing, indicating that the target-effector interactions were antigen specific and that there was little bystander killing in this system (Fig. 4B).

We examined the kinetic behavior of these CTL populations to determine the effect of activation with and without TCR engagement. There was a strong correlation between activation status and T cell displacement. Vector displacement, as calculated from position at time 0 and position at time 30 h, reflects the inherent traveling capacity of the T cell in an antigen-rich environment. Naïve cells moved an average of 17.5 µm, compared with 40.3 µm with activation by concavalin A. Activated T cells in the presence of cognate antigen travelled even further, averaging 65.3 µm for antigen-stimulated B6 CD8^+^ T cells and 74.4 µm for OT-1 T cells (Fig. 4C).

CD8^+^ T cell velocities similarly showed marked enhancement with activation, even without engagement of the T cell receptor. OT-1 T cells and OVA-stimulated B6 T cells averaged velocities of 251 and 243 nm.s^−1^ respectively. The average velocity of concavalin A stimulated T cells was 80% of that of antigen-specific T cells, compared with only 25% for naïve cells (Fig. 4D), suggesting that activation strongly enhanced CTL velocity even in the absence of activation of the T cell receptor.

These data suggest that CD8^+^ T cell activation enhances their intrinsic kinematic ability above the naïve state, and that these parameters may be further influenced by the presence of cognate antigen.

### CTL killing ability and kinematics are enhanced by cognate antigen

**Figure 5:**
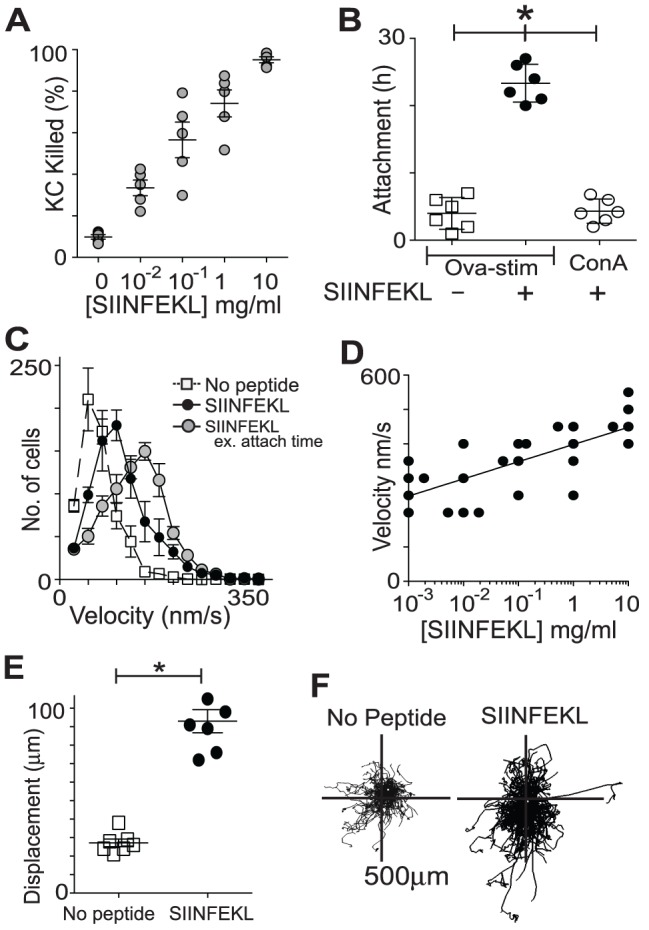
KC killing is dependent on antigen presentation and T cell activity. **A**. Antigen-specific CTL were co-cultured with B6 KC loaded with increasing concentrations of SIINFEKL peptide. Percentage of total KC killed during of imaging 30 hours was measured by fluormetric detection of caspase-3 activation. (N = 5, error bars SEM). **B**. Average duration of attachments of activated T cells, either antigen specific Ova-stim CTL or non-specifically activated ConA CTL, to targets with or without peptide. (N = 6, error bars SEM). **C**. Velocities of antigen-specific CTL added to target KC, with and without peptide loading, were calculated from 30 h tracks. Values were binned 25 nm.s^−1^ and averages of 5 experiments are shown. Error bars are SEM. **D**. Examination of the velocities of the effector T cells (from (A)) in response to different concentrations of peptide loaded onto target cells. Linear regression line shown. **E**. Average displacements of CTL in the presence or absence of cognate antigen. (N = 5, error bars SEM) **F**. Example of actual tracks of CTL cultured with target cells, with and without peptide, translated to a common starting point. (*p<0.05, *p<0.05 between indicated groups ANOVA.).

To determine if the strength of TCR signal affected the kinematics and target cell killing, we investigated the effect of varying the concentration of peptide presented on target cells. CD8^+^ T cells from OVA-immunised EGFP^+^B6 mice were harvested after short stimulation and added to cultured B6 KC loaded with SIINFEKL peptide in varying concentrations. Consistent with other published killing assays, CD8^+^ cells killed more effectively when target cells presented high levels of cognate antigen (Fig. 5A), reaching almost 100% target cell loss at 10 µg.ml^−1^ of SIINFEKL. We compared SIINFEKL antigen loading of KC to the effects of native expression of OVA protein from a transgene. When KC were derived from K5mOva transgenic mice that express OVA from the keratin-5 promoter, these were killed at 52% (+/− 22%) efficiency: comparable to B6-derived KC loaded with 0.1–1 µg.ml^−1^ of SIINFEKL (data not shown).

We examined the movement kinetics of effector cells incubated with target cells presenting cognate antigen. Co-cultures of CD8^+^ T cells and KC with and without antigen were imaged for 30 hours. As in the above experiments, movies were analysed by identifying EGFP^+^ cells <12 µm in size and tracks of their movement over time were created using analysis software. Velocities were calculated while cells were moving. That is, attached and arrested cells were given a speed of 0 once they attached and were no longer included in further calculations. Thus the track velocities at which T cells reached their targets, as well as the length of time they attached could each be determined independently.

In this assay involving primary activated CTL and primary KC, killing clearly required prolonged effector-target cell attachment (Fig. 5B). We found that in the absence of cognate peptide, CD8^+^ T cells did not make long attachments with targets. Concavalin A stimulated cells also did not make long attachments with SIINFEKL-loaded targets, suggesting that the attachment phase was reliant on the T cell receptor interaction with cognate antigen rather than on activation of CD8^+^ T cells *per se* (Fig. 5B).

Examination of CTL kinematics showed that without antigen present, there was a global slowing of CD8^+^ T cells relative to CD8^+^ T cells recognizing cognate antigen (Fig. 5C) (p<0.001). We found a direct linear correlation between increasing peptide concentration on target cells and average CTL velocity that had not peaked with 10 µg.ml^−2^ loading (Fig. 5D). These data indicate a close association between CD8^+^ T cell movement kinetics and the amount of antigen presented by target cells.

We determined the net displacement of T cells during the period of co-culture. Activated CD8^+^ T cells incubated with KC loaded with no antigen appeared to move within a limited region (Movies S2, S3). These effector cells evidenced small displacements indicating a restricted range of travel (Fig. 5F). By contrast, the average displacement of CTL in the presence of cognate antigen was almost 3 times higher (Fig. 5E, Movies S5, S6). Interestingly, examination of co-cultures treated with Z-DEVD-FMK showed no significant difference in T cell arrest time, displacement, track length or speed compared with untreated co-cultures although cell death was abrogated (data not shown).

Movement did occur after T cells were attached to target cells, but as KC moved minimally in these culture conditions, this made very little difference to the net displacement value of the T cells. KC moved slower than T cells. Thus, when we examined the velocities of T cells carefully excluding the portions of the tracks after attachment to target cells by manual selection, this showed that moving T cells were actually even faster than calculated by using the whole tract by software only.

### T cell populations with more EM cells kill more efficiently

When CD8^+^ T cells become activated, they undergo a series of subcellular changes including increased size, increased cytoplasm:nuclear ratio, increased production of granzymes, and increased polarity, among others – resulting in an increase in the cytolytic potential of these cells [12]. However, dynamic speed and travel patterns of activated CD8^+^ T cells may be predominantly influenced by the tissue environment and antigen expression by target cells rather than mechanisms intrinsic to the T cell. We investigated the kinematic behaviour of cytotoxic T cells in relation to their activation status.

**Figure 6:**
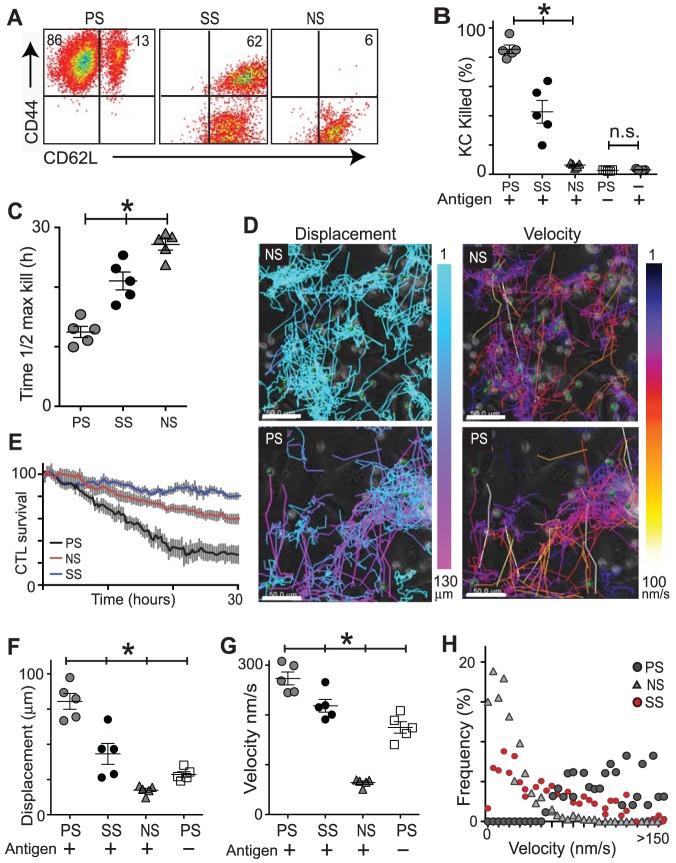
Kinematics of activated CD8^+^ T cells correlate with their cytotoxicity. CD8^+^ T cells were generated by prolonged stimulation (PS) of splenocytes from OVA-immunised mice with irradiated feeder cells pulsed with 0.1 mg.ml^−1^ peptide, or they underwent short stimulation (SS) by overnight culture with 1 mg.ml^−1^ of peptide; or they were not stimulated (NS) *in vitro* after immunization. These were added to cultured KC targets presenting SIINFEKL and imaged. **A**. T cells were isolated by FACS based on size, granularity, EGFP and CD8 expression. These CD8^+^ cells were then stained for CD44 and CD62L expression. **B**. Percentage of KC targets that were killed by each group of CTL over a 30 hour time-course is shown. PS T cells were also added to KC without peptide to show non-specific killing. Death rate of KC in monoculture is also shown. **C**. The time for each group of T cells to reach 50% of their total target cell death is shown. (N = 3 independent experiments) **D**. Examples of the movement tracks taken by CTL among the KC cells colour-coded for displacement and velocity. Scale bars, 50 µm. **E**. Survival of PS, NS and SS CTL over 30 hours of co-culture with antigen-loaded KC was determined from the number of GFP+ cells remaining as imaging progressed, as a percentage of the total number of GFP+ cells at time 0. Pooled data 5 experiments, error bars are SD. **F. & G**. Displacement and velocity of effector cell groups in culture with targets with and without antigen. (B, C, F, G N = 5, error bars SEM) **H**. Example of frequency plot of velocity values for the different populations of CTL. (*p<0.05 between indicated groups, n.s. not significant, ANOVA).

EGFP^+^B6 mice were immunized subcutaneously with OVA (and Quil A adjuvant). Mixed splenocytes were harvested 10 days after immunization and cultured in IL-2 and SIINFEKL for 3 days only, before CD8^+^ T cells were purified by FACS. This short stimulation generated a mixed population of T cells which were CD44^hi^CD62^hi^ (central memory phenotype) and CD44^low^CD62L^hi^ (naïve phenotype) [13]. To generate a population of high-affinity effector T cells, splenocytes were harvested and underwent prolonged stimulation by passage and culture with irradiated feeder splenocytes in the presence of low-dose (0.1 µM) SIINFEKL peptide and IL-2 for 6–8 weeks [14]. This yielded a population richer in CD44^hi^/CD62L^low^ (effector memory phenotype) cells and with no naïve T cells. CD8^+^ T cells were also harvested directly from immunised B6 mice without culture, yielding mostly a naïve, non-stimulated population (CD44^low^). Thus we generated CD8^+^ T cells with different possible cytotoxic capabilities [15] (Fig. 6A), which were then added to cultured KC targets presenting SIINFEKL.

We evaluated target cell killing by time-lapse microscopy. The non-stimulated CD8^+^ T cells (NS) killed very little, although the few activated cells (6%) in the co-culture were able to effect some killing above baseline. As expected, T cells from prolonged stimulation, which were expected to have a higher affinity, killed more targets (Fig. 6B), and in less time (Fig. 6C), than CTL from short stimulation indicating superior cytolytic efficiency of the cells from prolonged culture (Movies S2, S3). Without peptide loading of target cells, KC were not killed, even by highly activated T cells, indicating minimal bystander killing (Fig. 6B). There was a low level (<5%) of background cell death in this system as determined by culturing KC as monocultures on the same plate (Fig. 6B).

Highly active PS CTL displayed notably increased mortality over their less stimulated counterparts, with NS cells showing least mortality over 30 hours of co-culture (Fig. 6E). Over the 30 hours of imaging, the CTL were tracked by velocity and displacement (Fig. 6D). It was apparent that non-stimulated cells moved very short distances among the target KC on the plate, while short-stimulation of CTL markedly increased their displacement (Fig. 6F). It appeared that prolonged stimulation may have a further effect in increasing the range of travel. In the absence of cognate antigen, there was a small but significant increase in the displacement of stimulated cells compared with non-stimulated CTL. It appeared that activation of CTL increases their displacement which is, however, markedly dependent on expression of cognate antigen by target cells (Fig. 6F).

Activation of CTL by short stimulation increased track velocities from an average of 64 nm.s^−1^ in non-stimulated cells to 244 nm.s^−1^ (Fig. 6G, p<0.001). Cells stimulated by prolonged culture moved faster again, and were able to achieve increased velocities above non-stimulation levels – averaging 175 nm.s^−1^ – even in the absence of cognate antigen (Fig. 5F, p<0.001). CTL velocities seem to be determined by their activation status, with apparently less influence by expression of cognate antigen in a simple effector-target model.

While it appeared that the effect of increasing activation of CTL was directly correlated with increased kinematics, we were concerned that short stimulation had resulted in the production of a mixed population of lymphocytes. In fact, only 58% of these cells expressed the activation marker CD44^+^, leaving almost half with a naïve phenotype (Fig. 6A). Examination of the distribution of the velocities of our three populations of CTL revealed distinct motility behaviors. Non-stimulated cells were almost uniformly slow, with no cells moving more than a maximum velocity of 60 nm.s^−1^, while cells harvested after prolonged stimulation and more likely to be clonal were all moving at velocities of over 50 nm.s^−1^ (Fig. 6H). Consistent with their mixed composition of naïve and activated cells, the CTL from the short stimulation group displayed velocities including both very slow and very fast cells. It is possible that the cytotoxicity and kinematic behavior of this group of cells also reflects the average of activated and naïve cells (Fig. 6H).

We surmise from these data that the movement kinetics of CD8^+^ T cells are determined by inherent changes that occur with T cell activation and that these are further strongly influenced by the presence of cognate antigen.

### CTL ability to kill antigen-presenting targets is directly correlated with velocity

**Figure 7:**
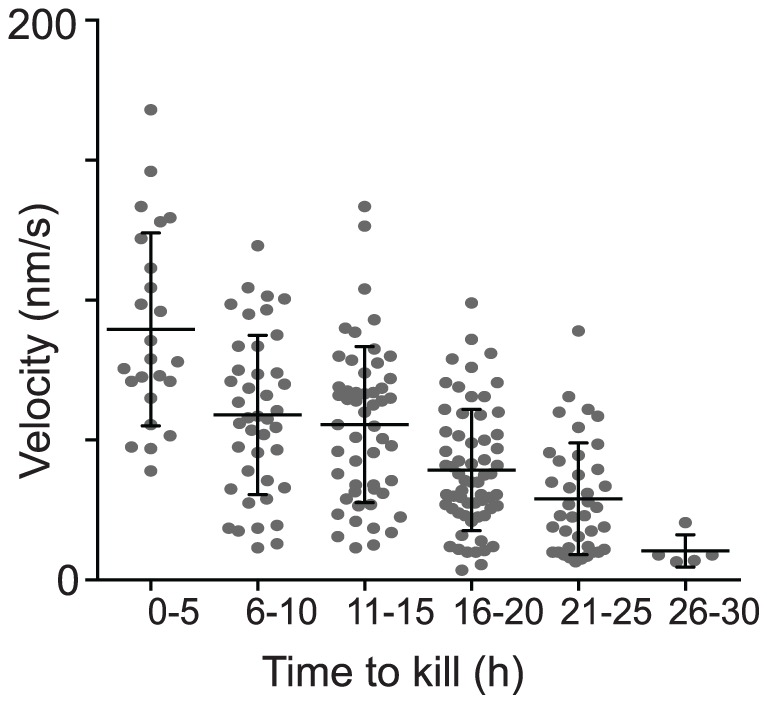
Velocity of a CTL directly correlates with its ability to kill. CTL were isolated from OVA-immunised mice and underwent short stimulation with antigen *in vitro* before adding to KC monolayers loaded with SIINFEKL and imaged for 30 h. Movies were examined in 5-hour time intervals to analyse target cell death in that time frame, and its killer CTL identified. If more than one CTL was attached to a dying KC, both were analysed. The average track velocity for each identified CTL in each time interval was determined. CTL which were not moving, were excluded. Pooled results N = 3 independent experiments. Mean and SD are shown.

The data presented so far have shown a positive association between increasing velocity and displacement and attachment times of CTL and their ability to kill. This has been strongly suggestive that increasing movement of a CTL indicates increased cytotoxic potential. We wished to investigate the cytotoxic behavior of each CTL in comparison to its kinematics. CTL were generated by short stimulation of CD8^+^ T cells from OVA-immunised B6 mice and these were added to KC loaded with SIINFEKL, imaging as above for 30 hours in the presence of FLIVO-red. At five-hour intervals, KC deaths were assessed and the lymphocyte responsible for each kill was identified by its unique identifier assigned by software (Fig. 7). The track velocities of each identified CTL were compared with the time at which each killed its targets.

There was an inverse correlation between the average velocity of each CTL and the time it took to kill a KC target (Fig. 7). The majority of kills occurred between 16–25 hours, as we showed in Fig. 3. These data indicate a direct relationship between the velocity of a CTL and the rate of target cell killing.

### CD8^+^ T cell death is related to antigen presentation by target cells

The unique design of this prolonged co-culture system revealed a very high rate of effector cell death, not usually described in other killing assays. We investigated this phenomenon. Naïve CD8 T cells, or CD8 T cells stimulated for a short time in culture, were harvested and added to KC presenting SIINFEKL peptide. As in the above experiments, co-cultures were grown in imaging media comprising supplement RPMI, supplemented SFM, IL-2, and FLIVO-red, and were kept at 37°C with 5% CO_2_.

**Figure 8:**
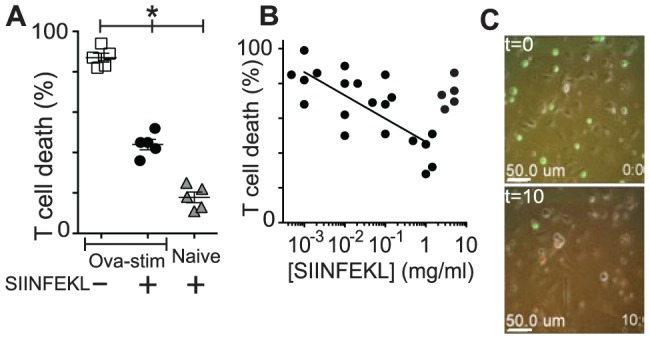
Co-culture conditions affect CD8^+^ T cell survival. CTL were isolated from OVA-immunised mice and were stimulated for a short time *in vitro* before adding to KC monolayers loaded with SIINFEKL and imaging for 20 hours. CTL death was determined by size (<10 µm), colour change (EGFP to red indicating loss of EGFP expression and caspase-3 activation) and morphology. **A**. T cell death in response to KC targets with 1 mg.ml^−1^ SIINFEKL and without peptide. (N = 5, error bars SEM, *p<0.05 between indicated groups) **B**. T cells were added to KCs loaded with increasing concentrations of SIINFEKL and CTL death over 30 hours was measured. (N = 5) **C**. CTL were added to KC monolayers loaded with 10 mg.ml^−1^ SIINFEKL. Still images from the beginning of co-culture (upper panel) and at 10 hours (lower panel) were taken by epifluorescent time-lapse microscopy.

As determined by size, and green-to-red colour change indicating loss of EGFP expression and caspase-3 activation, we found there was more CD8^+^ T cell death when CTL were co-cultured with KC not loaded with cognate antigen, compared with loading with 1 mg.ml^−1^ of SIINFEKL (Fig. 8A). However, when naïve cells were cultured with KC, they had very low death rates in similar conditions. When we examined the effect of antigen dose on T cell death rate, we found CTL death was inversely related to amount of antigen expressed, with the lowest death rate at our usual KC loading concentration of 1 mg.ml^−1^. At 10 mg.ml^−1^, CTL death rate rose sharply, in conjunction with markedly increased KC deaths (Fig. 8B). At such high doses of SIINFEKL, almost all T cells were dead by 10 hours (Movie S7, Fig. 8C). By comparison, in standard culture conditions, we are able to culture CTL in supplemented RPMI media with IL-2 for at least 24 hours with minimal cell loss.

Examination of these interactions revealed that during the process of target cell death, attached effector cells usually died. Thus at least at very high antigen concentrations, effector cell death was directly associated with a very high rate of early target cell death (Fig. 8B,C).

## Discussion

Tissue-resident CTL in skin appear to arrive early, persist, and are functionally responsive and protective upon re-exposure to antigen [16]. Given that their primary aim in inflammatory tissues is elimination of virally infected cells, lysis of cells presenting target antigen is crucial to their function. CD8^+^ T cell mediated apoptosis of keratinocytes is MHC class I restricted [17], and can be effected by processing and presentation of antigens by CD8^+^ T cells themselves [18]. We find that the process of killing of primary keratinocytes presenting physiological levels of non-self antigen is very slow, in contrast to tumor models and cell lines where killing has been reported within minutes to hours [19]. *In vivo*, there are very few studies able to quantify time required to effect CTL-mediated killing, likely because the process takes so many hours. However, recently, prolonged contact between effectors and targets has been shown to occur *in vivo* leading to target cell death [20]. It could be the case that KC as a cell type are inherently resistant to cell mediated cytolysis, reflecting the generally immune-tolerant behavior of skin. CD8^+^ T cell activity against different cells or tissues that express the same antigens can vary, reflecting tissue specific behavior of effectors [21]. CTL in some assays have been shown to kill KC as efficiently as they kill fibroblasts [1], but the differences between the kinetics of killing becomes clear using time-lapse imaging to assay target cell death rather than the traditional end-point chromium-release assays, which may be misleading in leaking chromium through permeabilized cells significantly earlier than caspase-3 activation and morphologic changes of apoptosis. We also note that CTL mediated killing was antigen dose dependent, stressing the importance of the careful selection of antigen dose in studying effector cell behavior. KC's inherent resistance to killing appears to emerge after target-effector attachment, which occurred early. As the attachment process requires interaction of the TCR with target antigen, it implies that deficient MHC class I dependent antigen processing is not the cause of the resistance to CTL-mediated killing in KC. We speculate that the formation of the immunological synapse or delivery of the killing signal may be less efficient in KC, particularly since we see enhanced killing by increasing the level of antigen presentation which has been shown to enhance the quality of immunological synapse formation [22]. Contact-dependent killing by NK cells has also been shown to vary according to target cell type. Interestingly, NK cells kill targets serially, but only the attachment time to the first target cell was dependent on target cell type, lending weight to the idea that the quality of the immunological synapse is responsible for the time from effector attachment to target kill [10].

We developed a co-culture system amenable to examining large numbers of interactions that could be analysed using large-capacity data handling hardware and software, to provide both quantitative and qualitative information regarding how primary antigen-specific T cells kill primary keratinocytes presenting antigen. While there have been studies that have examined trafficking of effector CD8^+^ T cells to target cells both *in vitro* and *in vivo*, the physiologic significance of the differences in kinematic behaviour of CTL seen in these studies remain unclear, although cytotoxic T cell function clearly requires cell motility. Studies examining the behavior of these cells in lymph nodes as they interacted with professional antigen presenting cells showed that cell-to-cell dwell time and the number of contacts made influenced the strength of the activation signal of the T cell [23]. In peripheral tissues and tumours, however, it is apparent that multiple variables including cytokine concentrations, target cell type, and the presence of other inhibitory and excitatory signals influence the behavior of cytotoxic T cells. Treg cells have been shown to both impede function and chemotaxis of CD8^+^ effector cells in skin [24]. Several *in vivo* studies have described T cell arrest in tissues in direct proportion to the amount of antigen present [7], [25], [26]. The function of these arrested cells is not clear. Enhancement of CD8^+^ T cell movement by inhibiting the PD1/PDL1 pathway led to reactivation and viral clearance from tissues [27]. However, in a tumour model, killing could be up regulated by slowing down T cells and decreasing their motility through tissue [28].

We demonstrate in this study that in a reductionist model, cytotoxic ability is associated with enhanced kinematics. T cells move further and faster both when they are activated and when there is more antigen present, both of which were directly associated with markedly more target cell killing. In addition, cells that killed earlier were more likely to be the faster ones (Fig. 5). The process of activation of CD8^+^ T cells lead to an increase in their kinematics, even in the absence of cognate antigen and without activation of the T cell receptor. However, the presence of cognate antigen on target cells increased both displacement and velocity of antigen-specific CTL. Interestingly, as CTL attached to their targets within 5–10 hours and then remained attached until killing at 15–25 hours post culture, most of the motility we saw was early in the culture prior to attachment of CTL to their targets, suggesting that it forms a part of target-seeking behavior. In contrast to these *in vitro* findings, *in vivo* imaging studies have generally described a slowing down of T cells in the presence of antigen [28] [29], although enhanced cytotoxicity has been also associated also with increasing kinematics [12], [27]. It appears from these conflicting data that the influences on the *in vivo* kinematics of CTL derive from factors other than just those in our reductionist 2-cell model. We speculate that the cytokine environment, and tissue oxygenation play important roles in determining CTL kinematics *in vivo*.

Enhanced kinematics are a result of a series of metabolic changes in the CTL that are likely triggered by engagement of the TCR or by cytokine secretion. In the present 2-cell culture model, the only possible sources of cytokine are KC or CTL cells themselves. As enhanced kinematics were seen before CD8^+^ T cells attached to KC to encounter antigen and as it is unlikely that increasing antigen loading on KC changes their cytokine secretion, we speculate that the drive for enhanced kinematics is likely to be from cytokine release by other T cells in the culture responding to higher levels of peptide expression. Also, possibly, the movement of CTL among antigen-presenting targets made a series of low-affinity contacts that progressively increased T cell velocity and displacement until a high quality effector-target synapse was achieved [30]. Similar behavior has been seen in lymph nodes, where each contact with antigen presenting cells lead to progressive enhancement of CTL activation [23]. *In vivo*, skin infiltrating T cells have an effector memory phenotype and thus have a high cytotoxic capability. Recently, it has been shown that activated T cells undergo distinct metabolic up-regulation compared with naïve cells [31]. This suggests that the limited trafficking of CD8^+^ T cells described previously in the epidermis [32] is due to down regulation of their cytotoxic function by factors in the local skin environment, and suggests that changes of inflammation in the skin markedly inhibit CTL function.

Utilising a reductionist approach to examining primary CD8^+^ cell motility allowed for examination of the fundamental biologic properties of activated CTL in the absence of tissue factors, and inflammation. Surprisingly, without danger signals, or inflammatory cytokines other than what the cells themselves produced, CTL displayed inherent motility upon activation. They notably travelled short distances in our assay compared with migration studies *in vivo*, even with activation and in the presence of antigen, around 90 - 100 µm per movie (Fig. 5E). This is not surprising considering that these cells encounter target cells expressing antigen very early, at which point they stop moving. Also the presence of tissue chemokines and cytokines likely affects their displacement *in vivo*. This study could not determine whether there is a difference in the behavior of cells that are “more” or “less” activated based on their activation phenotype, but as the cytotoxicity of CTL has been shown primarily to be dependent upon the affinity of the T cell receptor for its cognate antigen and as we show data that increasing presented antigen increases kinematics, we speculate that the enhanced kinematics are more likely to be an all-or-nothing switch which is then modulated by tissue and target cell factors.

Attachment time to target cells, possibly what is seen *in vivo* as arrested T cells, was not directly associated with increased killing as highly activated cells tended to kill their targets more quickly. However, attachment is clearly a function of the interaction between the CTL receptor and its cognate antigen, and is a determinant of the killing capacity of CTL independent of their other kinematics. Attachment time of CD8^+^ T cells to keratinocytes – likely what was seen *in vivo* as arrested T cells – was not directly associated with increased killing. *In vitro*, highly activated T cells tended to kill their targets more quickly. However, transient attachments were also seen in the absence of peptide and with naïve T cells, suggesting there is a minimal contact time required to achieve target death. This suggests a dual pattern of effector T cell arrest: prolonged contact resulting in target cell death when there was minimal antigen present, and short attachments leading to death only when there was a large amount of antigen presented by the targets, or when T cells were highly activated. These data imply that attachment, or T cell arrest, is a function of TCR and the quality of the immunological synapse made with target cells, which is consistent with reports showing TCR internalization in effectors to be a function of antigen presentation rather than T cell arrest duration *per se* [33].

CD8^+^ T cell death was seen to a surprising level in this assay. We speculate that this may be a phenomenon specific to the cell culture co-culture situation where CTL are grown in media not ideal for their development and they must compete with the much larger KC for nutrients, particularly since our assays continue for over 30 hours while most killing assays are less than 6 hours in duration. The lower metabolic activity of naïve T cells may protect them from competing with KC for nutrients. The dose-response nature of CTL death may be a reflection of KC killing at increasing antigen doses resulting in reduced cell numbers in culture. At very high levels of antigen, KC death was paralleled by T cell apoptosis, possibly by activation-induced cell death, a well-described process in effector T cells [34].

Intra-vital microscopy is usually limited by the short time during which images can be gathered and it is apparent from the current study that CTL killing of KC is a very long process. Thus despite the obvious limitations of an *in vitro* study of an *in vivo* process, valuable fundamental processes of CTL biology over periods of time may be investigated using the current approach. Here we utilise an *in vitro* reductionist technique for investigating the basic biological properties of target cell killing by CD8^+^ T cells. These data provide a fundamental framework for understanding the relationship between the kinematics of T cells *in vivo* and CTL function, and help to interpret the patterns of movement seen in intra-vital imaging studies.

## Supporting Information

Movie S1 CD8^+^ T cells require direct prolonged contact with target cells to kill KC CD8^+^ T cells were loaded with SIINFEKL and incubated with effector CD8^+^ T cells from EGFP^+^OT-1 mice. Images were taken by confocal microscopy every 12 minutes in 2 µm Z-sections and have been shown here as maximal intensity projections. Cell permeable indicator dye in the media fluoresces red upon activation of caspase-3. Effector cells are seen to attach to KC early in the incubation period. KC death is seen at 21 hours by colour change as caspase-3 is activated, and then by blebbing. Target cell death is seen by both morphological changes of apoptosis and colour change, and appears to be accompanied by death of the effector cell also.
(MOV)

Movie S2 Co-culture of effector memory phenotype cells and target cells results in rapid contact-dependent KC death. Effector memory phenotype CD8^+^ T cells were generated by prolonged stimulation with irradiated peptide-loaded feeder splenocytes and SIINFEKL, and then co-cultured with target KC presenting cognate peptide. Highly activated effector cells move rapidly among targets and form attachments to target cells resulting in rapid death.
(MOV)

Movie S3 Co-culture of effector memory phenotype cells and target cells leads to rapid death of both cell types. T cells in Movie S2 were identified by size and fluorescence and tracked over time. Tracks displaying 20 frame tails are displayed, and have been colour coded to indicate vector displacement length. Note short travel lengths and minimal displacement.
(MOV)

Movie S4 KC cultured without T cells show minimal death over 30 h. Caspase-3 indicator dye has been added to the culture. There is minimal cell death and minimal KC motility seen.
(MOV)

Movie S5 T cells move further, and attach for longer to KC loaded with peptide. Primary KC in culture were loaded with SIINFEKL, and co-cultured with EGFP^+^OT-1 T cells for 30 hours. Without peptide loading, KC interactions with effector cells are short. Effector cells move in a limited fashion and die within hours.
(MOV)

Movie S6 T cells move further, in co-culture with KC loaded with peptide. Effector T cells from Movie S5 were identified by size and fluorescence and tracked over time. Tracks displaying 20 frame tails are displayed, and have been colour coded to indicate displacement length. Note the much longer travel distances and displacement of these effectors.
(MOV)

Movie S7 Examples of Co-cultures. Co-culture of EGFP^+^OT-1 T cells and primary KC loaded with 1 µg.ml^−1^. Effector cells travel further and their interactions with target cells are longer.
(MOV)

Movie S8 Examples of Co-cultures with killing. In this example, a CTL initially samples the KC but does not attach and the CTL moves away. Another CTL attaches to the target and remains attached until apoptosis takes place, with both effector and KC dying.
(MOV)

Movie S9 Movie S8 showing only the red channel. Note colour change of KC and the numerous smaller T cells.
(MOV)

Movie S10 Movie S8 showing spot selection with manual correction. Dead T cells (red^+^, size 7 µm); EGFP^+^ T cells are green^+^, size 7 µm; Dead KC are denoted by purple spot, (red^+^, size 17 µm).
(MOV)
