# Correction: The Kinematics of Cytotoxic Lymphocytes Influence Their Ability to Kill Target Cells

**DOI:** 10.1371/journal.pone.0101381

**Published:** 2014-06-20

**Authors:** The PLOS ONE Staff

## Notice of Republication

This article was republished on June 2, 2014, to replace figures in the online version of the article that had lost color during the production process. The publisher apologizes for the errors. Please view this article again to download the correct figures.
